# Red and Processed Meat and Colorectal Cancer Incidence: Meta-Analysis of Prospective Studies

**DOI:** 10.1371/journal.pone.0020456

**Published:** 2011-06-06

**Authors:** Doris S. M. Chan, Rosa Lau, Dagfinn Aune, Rui Vieira, Darren C. Greenwood, Ellen Kampman, Teresa Norat

**Affiliations:** 1 Department of Epidemiology and Biostatistics, School of Public Health, Imperial College London, London, United Kingdom; 2 Biostatistics Unit, Centre for Epidemiology and Biostatistics, University of Leeds, Leeds, United Kingdom; 3 Division of Human Nutrition, Wageningen University, Wageningen, The Netherlands; Paris Institute of Technology for Life, Food and Environmental Sciences, France

## Abstract

**Background:**

The evidence that red and processed meat influences colorectal carcinogenesis was judged convincing in the 2007 World Cancer Research Fund/American Institute of Cancer Research report. Since then, ten prospective studies have published new results. Here we update the evidence from prospective studies and explore whether there is a non-linear association of red and processed meats with colorectal cancer risk.

**Methods and Findings:**

Relevant prospective studies were identified in PubMed until March 2011. For each study, relative risks and 95% confidence intervals (CI) were extracted and pooled with a random-effects model, weighting for the inverse of the variance, in highest versus lowest intake comparison, and dose-response meta-analyses. Red and processed meats intake was associated with increased colorectal cancer risk. The summary relative risk (RR) of colorectal cancer for the highest versus the lowest intake was 1.22 (95% CI  = 1.11−1.34) and the RR for every 100 g/day increase was 1.14 (95% CI  = 1.04−1.24). Non-linear dose-response meta-analyses revealed that colorectal cancer risk increases approximately linearly with increasing intake of red and processed meats up to approximately 140 g/day, where the curve approaches its plateau. The associations were similar for colon and rectal cancer risk. When analyzed separately, colorectal cancer risk was related to intake of fresh red meat (RR_ for 100 g/day increase_  = 1.17, 95% CI  = 1.05−1.31) and processed meat (RR _for 50 g/day increase_  = 1.18, 95% CI  = 1.10−1.28). Similar results were observed for colon cancer, but for rectal cancer, no significant associations were observed.

**Conclusions:**

High intake of red and processed meat is associated with significant increased risk of colorectal, colon and rectal cancers. The overall evidence of prospective studies supports limiting red and processed meat consumption as one of the dietary recommendations for the prevention of colorectal cancer.

## Introduction

Colorectal cancer is the third most frequently diagnosed cancer worldwide, accounting for more than one million cases and 600 000 deaths every year. Incidence rates are highest in North America, Western Europe, Australia/New Zealand, and in Asian countries that have experienced nutrition transition, such as Japan, Singapore, and North-Korea [1]. Incidence rates are stable or decreasing in long-standing economically developed countries, while they continue to increase in economically transitioning countries. Recent declines in mortality from colorectal cancer have been observed in North America and Japan, possibly due to primary prevention (surveillance and screening) and improved treatment [2]. Decreasing trends in colorectal cancer mortality have also been observed in most Western European countries [3].

The role of environmental and lifestyle factors on colorectal carcinogenesis is indicated by the increase in colorectal cancer incidence in parallel with economic development and adoption of a western lifestyle [4], as well as by the results of migration studies that demonstrate a greater lifetime incidence of colorectal cancer among immigrants to high-incidence, industrialized countries compared to residents remaining in low-incidence countries [5]. Screening and surveillance of adenomatous polyps, a precursor of colorectal cancer, is currently the cornerstone for primary prevention of colorectal cancer [6]. However, understanding the role of environmental factors in colorectal carcinogenesis may inform additional primary prevention strategies that can further reduce risk.

Several plausible biological mechanisms have been suggested to explain the association of red and processed meats with colorectal cancer [7]–[9]. These include the potential mutagenic effect of heterocyclic amines (HCA) contained in meat cooked at high temperature [10], but this is not specific of red and processed meats since HCA's are also formed in poultry. A second mechanism involves endogenous formation in the gastrointestinal tract of *N*-nitroso compounds, many of which are carcinogenic. Red meat but not white meat intake shows a dose–response relation with the endogenous formation of nitroso compounds in humans [11]. This has been explained by the abundant presence of heme in red meat that can readily become nitrosylated and act as a nitrosating agent [12], [13]. Nitrites or nitrates added to meat for preservation could increase exogenous exposure to nitrosamines, *N*-nitroso compounds, and their precursors; meats cured with nitrite have the same effect as fresh red meat on endogenous nitrosation [14].

In the 2007 World Cancer Research Fund and American Institute of Cancer Research (WCRF/AICR) report “Food, Nutrition, Physical Activity, and the Prevention of Cancer: a Global Perspective”, an international panel of experts based on an extensive review of the existing evidence concluded that high intake of red and processed meat convincingly increases the risk of colorectal cancer [15]. However two recent reviews of prospective studies concluded that the available epidemiologic evidence is not sufficient to support an independent positive association between red meat or processed meat consumption and colorectal cancer, because the likely influence of confounding by other dietary and lifestyle factors, the weak magnitude of the observed association, and its variability by gender and cancer subsite [16], [17]. Indeed, a positive association has been suggested in most but not all epidemiologic studies [15], and in some well conducted prospective studies, the association between red and processed meat and colorectal cancer was attenuated after better adjustment for potential confounders [18].

Since then, new results from ten prospective studies [19]–[28] have been published. This included studies in Asian populations [20], [25], [27], [28], a Canadian breast cancer screening cohort [24], a US multi-ethnic cohort [26], and four American cohorts [19], [21]–[23].We have focused our review on prospective studies, because case-control studies are more liable to recall and selection bias, and randomized controlled trials on red and processed meats and colorectal cancer are considered not feasible. The data on the relation of red and processed meats and colorectal cancers are summarized in highest versus lowest meta-analyses. Because stronger causal inference can be drawn from dose-response associations, we also conduct linear dose-response analyses. None of the previous meta-analyses have examined the shape of the dose-response relationship; we further explore whether there is a non-linear dose-response relationship between red and processed meats intake and colorectal cancer risk.

## Methods

### Data sources and search

We performed a systematic search for publications on red and processed meat and colorectal cancer in Pubmed, without any language restriction from 1966 to 31 March 2011, using the search strategy implemented for the WCRF/AICR report [15] (Text S1). The medical subject headings and text words covered a broad range of factors on foods and foods components, physical activity, and anthropometry. We also hand-searched reference lists from retrieved articles, reviews, and meta-analysis papers. The complete protocol and full search strategy used is available at http://www.dietandcancerreport.org/cu/ [29].

### Inclusion criteria and data extraction

Studies were included if they reported estimates of the association of red meats, processed meats, or both with colorectal, colon, or rectal cancer risk. “Red meat” was described in most studies as the intake of beef, veal, pork, mutton and lamb. “Processed meat” was defined as the total intake of ham, bacon, sausages, cured or preserved meats. Here, “red and processed meats” is used to denote the food item that includes both “red meats” and “processed meats” into a single item in the studies identified in the search.

To be included in the dose-response meta-analyses, the numbers of cases and the denominators in the cohort studies or the information required to derive them using standard methods [30] had to be reported. Other data extracted were study characteristics, cancer outcome, description of meat item, method of dietary assessment, and adjustment factors. When multiple articles on the same study were found, the selection of results for the meta-analysis was based on longer follow-up, more cases identified, and completeness of the information required to do the meta-analysis.

The search, study selection, and data extraction was conducted by several reviewers (led by EK) at Wageningen University, The Netherlands up to June 2006, and by two reviewers (DSMC and RL, led by TN) at Imperial College London from June 2006 to March 2011.

### Statistical analysis

Relative risk estimates were pooled using fixed-effects and random-effects models. We present the results from the random-effects meta-analysis that accounts for between-study heterogeneity [31] unless otherwise specified. We conducted meta-analyses for red and processed meats, combined and separately, using the description of the meat items given in the articles. In highest versus lowest meta-analyses (the comparison of the highest intake level to the lowest intake level), the relative risk (RR) estimate from each study was weighted by the inverse of the variance to calculate summary relative risks (RR) and 95% confidence intervals (CI). In linear dose-response meta-analyses, we pooled the relative risk estimates per unit of intake increase (with its standard error) reported in the studies, or computed by us from the categorical data using generalized least-squares for trend estimation [32]. When intake was expressed in “times” or “servings of intake”, we converted it into grams (g) using 120 g as a standard portion size for red and processed meat combined and for red meat, and 50 g was assumed as standard portion size for processed meat, as in the WCRF/AICR report [15]. Means or medians of the intake categories were used when reported in the articles; if not reported, midpoints were assigned to the relative risk of the corresponding category. Zero consumption was used as boundary when the lowest category was open-ended and when the highest category was open-ended, we used the amplitude of the lower nearest category. For studies reporting intakes in grams/1000 kcal/day [22], [26], [33], the intake in grams/day was estimated using the average energy intake reported in the article. When a study provided results by gender, we first pooled these estimates using a fixed-effects model and included the pooled value in the meta-analysis. One study provided results for distal and proximal colon cancer [34] and we derived the relative risk for colon cancer using the same procedure. We also conducted meta-analyses stratified by cancer sub-site, gender, and geographic area. Dose-response relationships were expressed per increment of intake of 100 grams per day for red and processed meat, and 50 grams per day for processed meat as in previous meta-analyses [15], [35].

To assess heterogeneity, we computed the Cochran *Q* test and *I^2^* statistic [36]. Sources of heterogeneity were explored in stratified analysis and by linear meta-regression, with gender, geographic area, year of publication, length of follow-up, and adjustment for confounders as potential explanatory factors. We also explored if heterogeneity of results was explained by the studies in which a standard portion size was used to convert times/servings per day to grams per day, and by method of dietary assessment. Small study and publication bias were examined visually in funnel plots for asymmetry and by Egger's test [37]. The influence of each individual study on the summary RR was examined by excluding each study in turn from the pooled estimate [38].

We further examined the potential non-linear dose-response relationship between red and processed meats and colorectal cancer using fractional polynomial models [39]. We determined the best fitting second order fractional polynomial regression model, defined as the one with the lowest deviance. Non-linearity was tested using the likelihood ratio test [40]. All analyses were conducted using Stata version 9.2 (StataCorp. 2005. *Stata Statistical Software: Release 9*. College Station, TX: StataCorp LP). P<0.05 was considered statistically significant.

## Results

### Results of search and study selection

**Figure 1:**
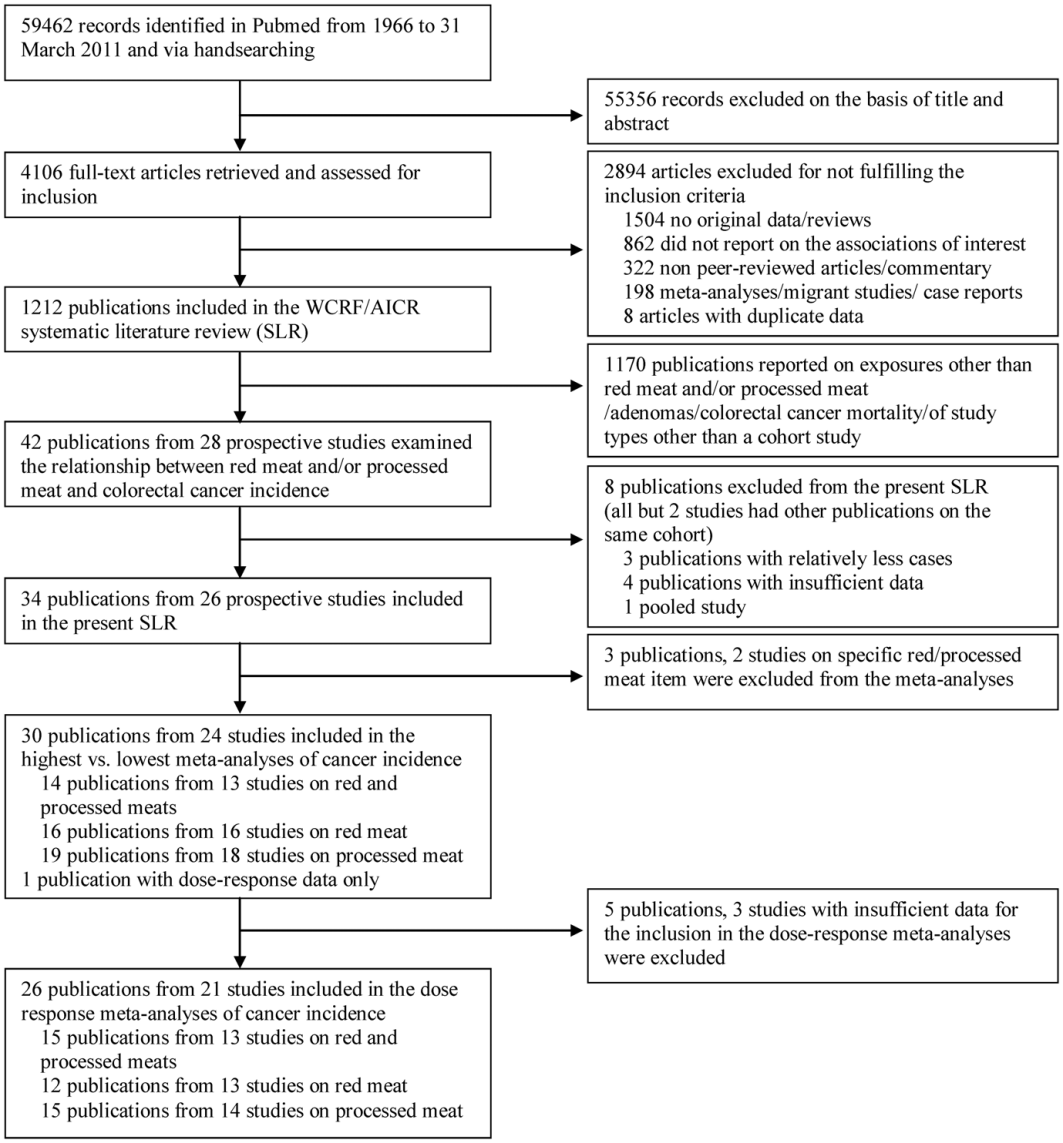
Flow diagram of systematic literature search on red and processed meat and the risk of colorectal cancer.

Forty-two articles from 28 prospective studies that examined the relationship of red and/or processed meat intakes and colorectal, colon, and rectal cancer incidence were identified (Figure 1). Eight articles were excluded [41]–[48] because other articles of the same cohort studies with more cases [49]–[51] or with information required in the meta-analysis were already included [18], [52], [53]. We could not include the UK Dietary Cohort Consortium [42], as data from two of the seven component cohorts were in other cohort consortium that was included in the meta-analysis because it had more cancer cases [50]. Hence, 24 prospective studies (2 case-cohort, 3 nested case-control and 19 cohort studies) were included in the highest versus lowest meta-analyses, of which 21 studies provided enough information to be included in the dose-response meta-analyses.

### Characteristics of the study cohorts

There were 13 cohorts of men and women, three male cohorts, and eight female cohorts. Twelve studies were from North-America, including a multiethnic cohort. The European Prospective Investigation into Cancer and Nutrition (EPIC) study involved ten European countries. The remaining were two studies each from Finland, the Netherlands, and Japan, and one study each from Australia, Canada, Sweden, China, and a Singaporean study with Chinese participants.

In all studies, relative risk estimates were adjusted for age and sex, and all except two adjusted for total energy intake. More than half of the study results were adjusted for body mass index (BMI), smoking, alcohol consumption, or physical activity, close to half controlled for dairy food or calcium intake, social economic status, family history of colorectal cancer, or plant food or folate intake. In some studies, the estimates were controlled for use of non-steroidal anti-inflammatory drugs, fish or white meat intake. The main characteristics of studies included in the dose-response meta-analysis are shown in table s1. Study results not included in the dose-response meta-analysis are detailed in table S2.

### Total red and processed meats

**Figure 2:**
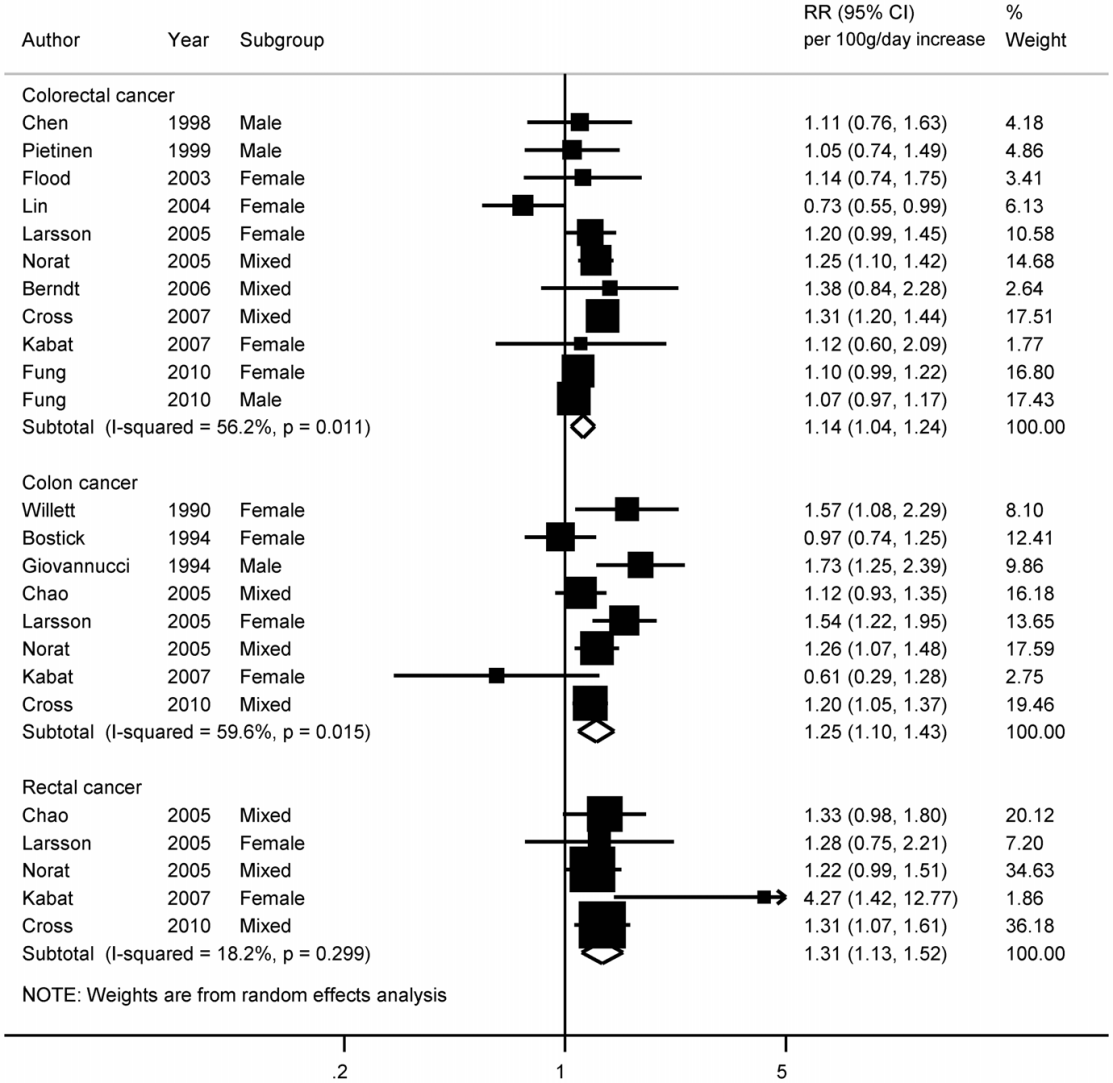
Dose-response meta-analyses of total red and processed meats consumption and the risk of colorectal, colon and rectal cancers. References: Chen, 1998 [51]; Pietinen, 1999 [57]; Flood, 2003 [33]; Lin, 2004 [56]; Larsson, 2005 [34]; Norat, 2005 [50]; Berndt, 2006 [19]; Cross, 2007 [22]; Kabat, 2007 [24]; Fung, 2010 [23]; Willett, 1990 [58]; Bostick, 1994 [52]; Giovannuccci, 1994 [55]; Chao, 2005 [54]; Cross, 2010 [21].

Thirteen prospective studies[19], [21]–[24], [33], [34], [50]–[52], [54]–[58] on total red and processed meats and colorectal cancer incidence were included in the highest versus lowest and dose-response meta-analyses. In highest versus lowest meta-analyses, red and processed meats intake was significantly related to an increased risk of colorectal (RR _highest vs lowest_  = 1.22, 95% CI  = 1.11−1.34), colon (RR_ highest vs lowest_  = 1.19, 95% CI  = 1.06−1.34), and rectal cancer (RR_ highest vs lowest_  = 1.51, 95% CI  = 1.31−1.75) (Table1). The mean values of the highest category of red and processed meats intake in the studies ranged from 46 to 211 grams per day. In dose-response meta-analysis, red and processed meats intake was positively related to colorectal cancer risk (RR _for 100 g/day increase_  = 1.14, 95% CI  = 1.04−1.24) (11 studies, 11358 cases) (Table1) (Figure 2). There was evidence of moderate heterogeneity between studies (*I^2^* = 56%, P = 0.01), that was significantly explained by intake unit conversion in the meta-regression (P = 0.00). Studies that required conversion from times or servings to grams per day [23], [51], [56] were significantly associated with a lower summary estimate than studies that did not require the conversion [19], [22], [24], [33], [34], [50], [57].

**Table 1:** Summary relative risks of meta-analyses of red and processed meats, red meat and processed meat, and colorectal cancer for all studies and by subgroups.

	Red and processed meats			Red meat			Processed meat		
	Pooled RR (95% CI)*,P value	n	Heterogeneity *I^2^*, P value	Pooled RR (95% CI)*,P value	n	Heterogeneity*I^2^*, P value	Pooled RR (95% CI)*,P value	n	Heterogeneity *I^2^*, P value
Dose-response meta-analysis Per 100 g/day				Per 100 g/day			Per 50 g/day		
All studies									
Colorectal cancer	1.14 (1.04–1.24), 0.00	11	56%, 0.01	1.17 (1.05–1.31), 0.01	8	0%, 0.48	1.18 (1.10–1.28), 0.00	9	12%, 0.33
Colon cancer	1.25 (1.10–1.43), 0.00	8	60%, 0.02	1.17 (1.02–1.33), 0.02	10	0%, 0.65	1.24 (1.13–1.35), 0.00	10	0%, 0.65
Proximal colon cancer	1.11 (0.88–1.40), 0.37	2	0%, 0.67	–	1	–	1.12 (0.81–1.56), 0.49	2	0%, 0.64
Distal colon cancer	1.22 (0.62–2.38), 0.57	2	90%, 0.00	–	1	–	1.41 (0.93–2.14), 0.10	2	0%, 0.69
Rectal cancer	1.31 (1.13–1.52), 0.00	5	18%, 0.30	1.18 (0.98–1.42), 0.08	7	0%, 0.67	1.12 (0.99–1.28), 0.08	8	0%, 0.56
By gender									
Men†									
Colorectal cancer	1.07 (0.98–1.16), 0.14	3	0%, 0.97	1.28 (0.49–3.35), 0.61	2	64%, 0.09	1.11 (0.86–1.44), 0.42	2	35%, 0.22
Colon cancer	1.41 (0.98–2.03), 0.07	2	71%, 0.06	1.06 (0.75–1.50), 0.73	2	0%, 0.98	1.64 (0.94–2.84), 0.08	3	72%, 0.03
Women									
Colorectal cancer	1.05 (0.90–1.23), 0.51	5	49%, 0.10	1.05 (0.78–1.42), 0.73	3	22%, 0.28	1.09 (0.89–1.33), 0.43	4	0%, 0.48
Colon cancer	1.15 (0.87–1.52), 0.33	5	70%, 0.01	1.16 (0.84–1.61), 0.37	5	28%, 0.23	1.33 (1.07–1.66), 0.01	5	0%, 0.75
Rectal cancer	2.12 (0.66– 6.77), 0.21	2	73%, 0.05	0.90 (0.60–1.35), 0.60	3	0%, 0.86	0.94 (0.62–1.44), 0.79	2	0%, 0.89
By geographic area									
Europe									
Colorectal cancer	1.22 (1.10–1.35), 0.00	3	0%, 0.65	1.23 (1.08–1.40), 0.00	5	0%, 0.63	1.13 (1.04–1.24), 0.01	4	0%, 0.73
Colon cancer	1.37 (1.13–1.66), 0.00	2	47%, 0.17	1.29 (1.08–1.54), 0.01	3	0%, 0.37	1.18 (1.05–1.33), 0.01	3	0%, 0.99
Rectal cancer	1.23 (1.01–1.50), 0.04	2	0%, 0.87	1.20 (0.95–1.50), 0.13	3	0%, 0.74	1.07 (0.92–1.25), 0.37	3	0%, 0.70
North America									
Colorectal cancer	1.11 (0.98–1.25), 0.09	8	66%, 0.00	–	1	–	1.21 (1.04–1.42), 0.01	4	11%, 0.34
Colon cancer	1.20 (1.01–1.43), 0.04	6	62%, 0.02	1.11 (0.86–1.44), 0.43	4	0%, 0.75	1.27 (1.10–1.47), 0.00	5	0%, 0.74
Rectal cancer	1.44 (1.05–1.96), 0.02	3	54%, 0.12	0.93 (0.54–1.60), 0.80	2	0%, 0.95	1.19 (0.92–1.55), 0.18	4	0%, 0.44
Asia-Pacific									
Colorectal cancer	–	0	–	1.01 (0.69–1.48), 0.96	2	56%, 0.13	–	1	–
Colon cancer	–	0	–	0.94 (0.69–1.27), 0.67	3	0%, 0.81	1.91 (1.05–3.48), 0.04	2	27%, 0.24
Rectal cancer	–	0	–	1.16 (0.57–2.39), 0.68	2	60%, 0.11	–	1	–
Highest versus lowest meta-analysis									
Colorectal cancer	1.22 (1.11–1.34), 0.00	10	14%, 0.31	1.10 (1.00–1.21), 0.04	12	22%, 0.22	1.17 (1.09–1.25), 0.00	13	6%, 0.39
Colon cancer	1.19 (1.06–1.34), 0.00	8	20%, 0.27	1.18 (1.04–1.35), 0.01	10	0%, 0.70	1.19 (1.11–1.29), 0.00	11	0%, 0.88
Proximal colon cancer	1.13 (0.97–1.32), 0.11	5	0%, 0.85	1.13 (0.83–1.54), 0.43	2	0%, 0.83	1.04 (0.90–1.20), 0.59	5	0%, 0.78
Distal colon cancer	1.36 (0.93–1.98), 0.11	5	71%, 0.01	1.57 (0.98–2.49), 0.06	2	53%, 0.15	1.20 (1.01–1.44), 0.04	5	0%, 0.41
Rectal cancer	1.51 (1.31–1.75), 0.00	6	0%, 0.76	1.14 (0.83–1.56), 0.43	7	38%, 0.14	1.19 (1.02–1.39), 0.03	9	20%, 0.27

*RR – relative risk; CI – confidence interval; n – number of studies †There is only one male cohort reported results on rectal cancer.

Intake of red and processed meats was significantly associated with an increased risk of colon cancer (RR _for 100 g/day increase_  = 1.25, 95% CI  = 1.10−1.43) (8 studies, 5426 cases), with significant heterogeneity between studies (*I^2^* = 60%, P = 0.02). Meta-regression analysis showed that studies adjusted for age and energy only [55], [58] reported stronger associations than the more adjusted studies [9], [21], [24], [34], [52], [54] (P = 0.03). Red and processed meats intake was significantly associated with rectal cancer (RR _for 100 g/day increase_  = 1.31, 95% CI  = 1.13−1.52) (5 studies, 2091 cases). In influence analysis, the statistical significance of the associations with colorectal, colon, and rectal cancers remained when each study was excluded in turn.

**Figure 3:**
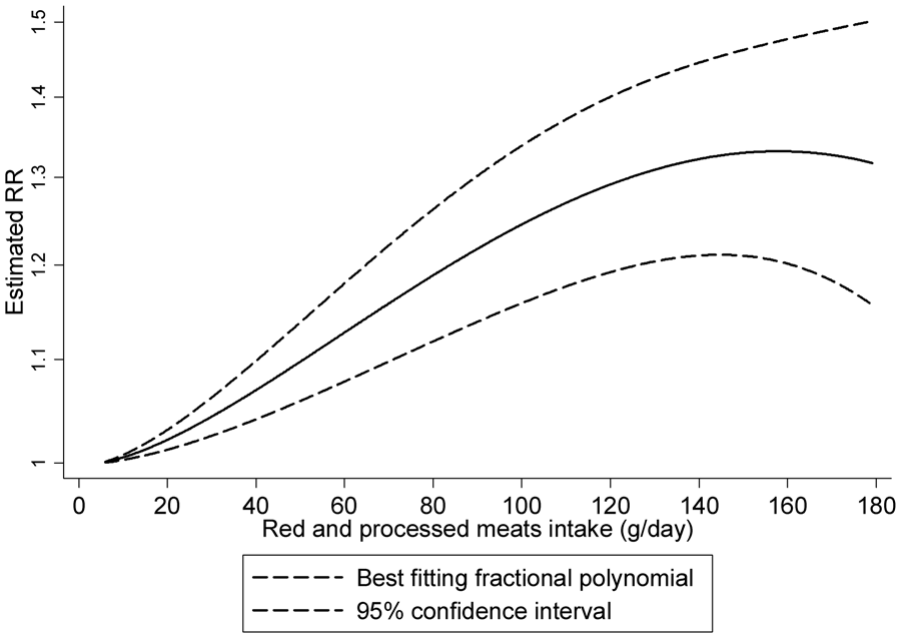
Non-linear dose-response meta-analysis of red and processed meats consumption and the risk of colorectal cancer.

There was evidence of a non-linear association of red and processed meats and colorectal cancer (P = 0.03). Visual inspection of the curve (Figure 3) suggests that the risk increases linearly up to approximately 140 g/day of intake. Above that intake level, the risk increase is less pronounced.

No significant associations were observed for proximal and distal colon cancers in the meta-analysis of the two [34], [54] out of the five studies [21], [34], [50], [54], [55] identified in the search (Table 1).

### Red meat

Sixteen prospective studies on red meat and colorectal cancer could be included in the highest versus lowest meta-analyses [18], [20], [21], [25]–[27], [34], [50], [52], [53], [57], [59]–[63]. From these, four articles could not be included in the dose-response meta-analyses because they did not provide sufficient data [20], [21], [59], [62].

**Figure 4:**
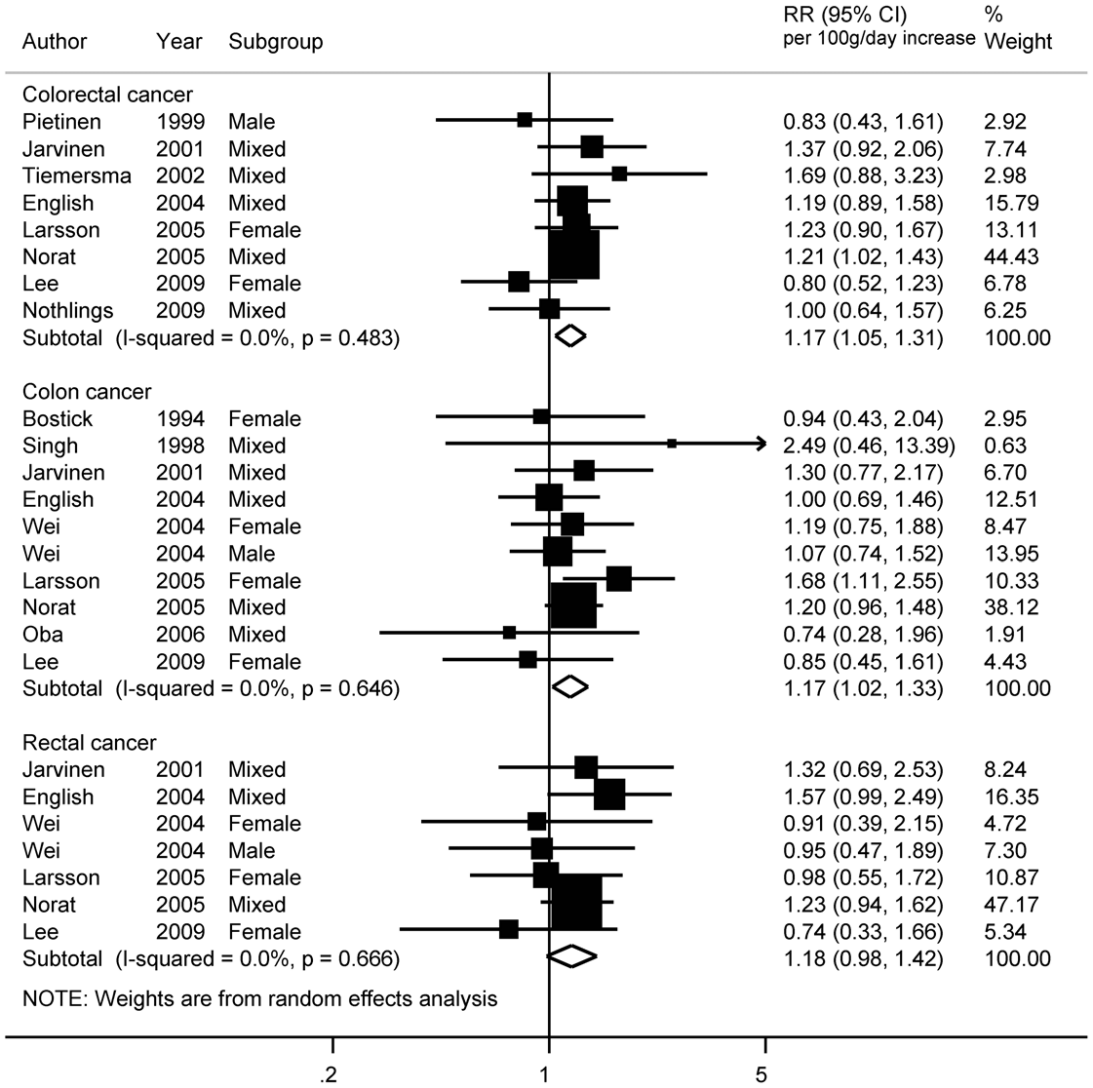
Dose-response meta-analyses of red meat consumption and the risk of colorectal, colon and rectal cancers. References: Pietinen, 1999 [57]; Jarvinen, 2001 [61]; Tiemersma, 2002 [63]; English, 2004 [60]; Larsson, 2005 [34]; Norat, 2005 [50]; Lee, 2009 [25]; Nothlings, 2009 [26]; Bostick, 1994 [52]; Singh, 1998 [53]; Wei, 2004 [18]; Oba, 2006 [27]

The summary RRs for the highest versus lowest red meat intake comparison were 1.10 (95% CI  = 1.00−1.21), 1.18 (95% CI  = 1.04−1.35), and 1.14 (95% CI  = 0.83−1.56) for colorectal, colon, and rectal cancer respectively (Table 1). The mean of the highest category of red meat intake ranged from 26 to 197 grams per day in the studies. In dose-response meta-analyses, red meat was statistically significantly associated with increased risk of colorectal (RR _for 100 g/day increase_  = 1.17, 95% CI  = 1.05−1.31) (8 studies, 4314 cases) and colon cancer (RR_ for 100 g/day increase_  = 1.17, 95% CI  = 1.02−1.33) (10 studies, 3561 cases) (Table 1) (Figure 4). No significant association was observed with rectal cancer (RR_ for 100 g/day increase_  = 1.18, 95% CI  = 0.98−1.42) (7 studies, 1477 cases). Influence analyses did not suggest strong influence from any of the individual studies on the summary estimates.

For proximal and distal colon cancers, no association was observed when combining the two studies identified [34], [50] (Table 1).

### Processed meat

**Figure 5:**
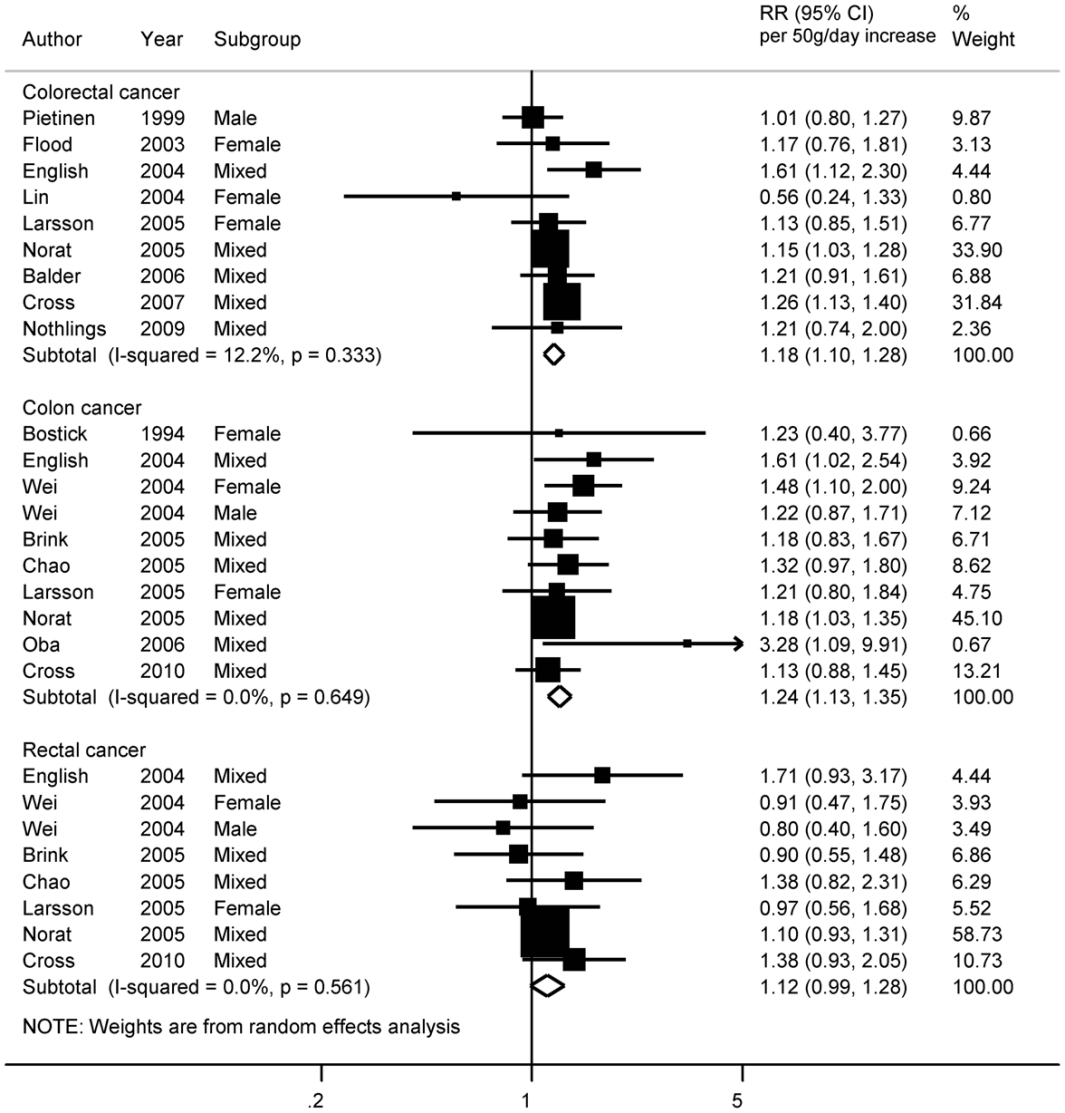
Dose-response meta-analyses of processed meat consumption and the risk of colorectal, colon and rectal cancers. References: Pietinen, 1999 [57]; Flood, 2003 [33]; English, 2004 [60]; Lin, 2004 [56]; Larsson, 2005 [34]; Norat, 2005 [50]; Balder, 2006 [64]; Cross, 2007 [22]; Nothlings, 2009 [26]; Bostick, 1994 [52]; Wei, 2004 [18]; Brink, 2005 [49]; Chao, 2005 [54]; Oba, 2006 [27]; Cross, 2010 [21].

Eighteen studies were included in the highest versus lowest meta-analyses [18], [20]–[22], [26]–[28], [33], [34], [49], [50], [52], [54], [56], [57], [60], [62], [64], [65]. Processed meat intake was significantly related to the risk of colorectal (RR _highest vs lowest_  = 1.17, 95% CI  = 1.09−1.25), colon (RR_ highest vs lowest_  = 1.19, 95% CI  = 1.11−1.29), and rectal cancer (RR_ highest vs lowest_  = 1.19, 95% CI  = 1.02−1.39) (Table 1). The mean of the highest category of processed meat intake ranged from 16 to 122 grams per day in the studies. Four studies could not be used to derive dose-response estimates [20], [28], [62], [66]. The summary RR for every 50 g/day increase in processed meat was 1.18 (95% CI  = 1.10−1.28) (9 studies, 10863 cases) for colorectal cancer and 1.24 (95% CI  = 1.13−1.35) (10 studies, 6727 cases) for colon cancer (Table 1) (Figure 5). For rectal cancer, no significant dose-response association was observed (RR _for_ _50 g/day increase_  = 1.12, 95% CI  = 0.99−1.28) (8 studies, 2565 cases). Influence analyses did not suggest strong influence from any of the individual studies on the summary estimates.

No significant associations were observed for proximal and distal colon cancers in the meta-analysis of the two [34], [54] out of five studies [21], [28], [34], [50], [54] identified in the search (Table 1).

### Small study or publication bias

In the analyses, no evidence of small study or publication bias was detected by visual inspection of the funnel plots. P for Egger's test ranged from 0.13 to 0.98 in the different analyses. The only evidence of publication bias was in the studies on processed meat and colon cancer, which suggested small studies with inverse association are missing (Egger's test P  = 0.06).

### Subgroup analyses

Table 1 shows the results of the dose-response meta-analyses by gender and geographic area. In most strata the number of studies was low and in some there was significant evidence of heterogeneity. Stratified analysis did not suggest any difference across gender. The association between red meat and colon cancer tended to be stronger in European studies (RR_ for 100 g/day increase_  = 1.29, 95% CI  = 1.08−1.54) (3 studies, 1307 cases) compared to the North American (RR _for 100 g/day increase_  = 1.11, 95% CI  = 0.86−1.44) (4 studies, 1476 cases) and Asia-Pacific studies (RR _for100 g/day increase_  = 0.94, 95% CI  = 0.69−1.27, P  = 0.67) (3 studies, 732 cases).

## Discussion

### Principal findings

The accumulated evidence from prospective studies supports that red and processed meats intake is associated with increased risk of colorectal, colon, and rectal cancers. The risk increase in colorectal cancer estimated in linear dose-response models was 14% for every 100 g/day increase of total red and processed meats, 25% in colon cancer, and 31% in rectal cancer. These results are consistent with those of the highest versus lowest meta-analyses. In non-linear models, colorectal cancer risk appears to increase almost linearly with increasing intake of red and processed meats up to approximately 140 g/day. Above this level, the risk increase is less pronounced.

Red meat intake (assessed separately from processed meat) was associated with increased risk of colorectal and colon cancers, but the association with rectal cancer was not statistically significant. Similarly, processed meat intake was related with risk of colorectal and colon cancers, but not with rectal cancer. The lack of association with rectal cancer is in contrast with the results observed when red and processed meats were combined into a single food item, where similar associations with colon and rectal cancers were observed. This may be due to a lower number of studies in the analyses of rectal cancer than in those of colorectal and colon cancers.

Our estimates are consistent with those reported in the 2007 WCRF/AICR expert report [15], where the risk increase of colon cancer was 37% for every 100 g/day increase in red and processed meats, and the risk increase of colorectal cancer was 29% for every 100 g/day increase in red meat, and 21% for every 50 g/day increase in processed meat.

### Selective reporting or publication bias

Some articles [20], [28], [59], [62], [66] could not be included in the dose-response meta-analysis because of insufficient information, but the dose-response meta-analyses were consistent with the highest versus lowest meta-analyses that included these studies that suggests that the exclusions from the dose-response meta-analyses did not bias our results. Two cohort studies could not be included in the meta-analysis. These studies reported positive but non-significant associations between fried sausage [67] and pork [68] and colon cancer. We could not include the results of the UK Dietary Cohort Consortium [42] that reported no association of red and/or processed meat and colorectal cancer in a pooled analysis of seven prospective studies with 579 colorectal cancer cases. The two largest cohorts – EPIC-Norfolk and EPIC-Oxford, participating in this consortium were included in our meta-analyses (Norat et al. EPIC); whereas individual results from the remaining five studies were not available. Our results are not in agreement with a preliminary analysis of 14 prospective studies with 7743 colorectal cancer cases from the Pooling Project of Prospective Studies of Diet and Cancer, that reported no association between red meat and processed meat and colorectal cancer (Cho and Smith-Warner. Proceedings of the American Association for Cancer Research. 2004; volume 45; abstract #491).

No evidence of publication bias emerged from visual inspection of funnel plots and Egger's tests in the analyses conducted, except for processed meat and colon cancer where there was a suggestion of small studies with inverse association missing. Since larger studies in the analysis have produced consistent results, it is unlikely for the missing studies to affect the association observed.

### Exploration of heterogeneity

There was evidence of heterogeneity between studies on red and processed meats and colorectal cancer, that was significantly explained by intake unit conversion in the meta-regression analysis. The summary risk estimate was lower in the studies for which we used a standard portion size in the unit conversion, compared to other studies. The approximation may have attenuated the association, and the real association may be stronger than showed in our estimates.

Meta-regression analysis indicated that level of adjustment partially explained the heterogeneity between studies on colon cancer. Studies adjusted for age and energy only (Nurses' Health Study - NHS [58] and Health Professional Follow-up Study - HPFS [55] showed a stronger association than studies with higher level of adjustments. However, after the exclusion of the studies adjusted only for age and energy intake from the analysis, moderate unexplained heterogeneity persisted. In a more recent article on the NHS and the HPFS, the associations of red meat and processed meat and colon cancer were attenuated after better adjustment for confounders and longer follow-up [18]. Nevertheless, in another recent article on the NHS, women who consumed one serving of red or processed meat daily for 40 years had a 20% increased risk of colon cancer compared with women who did not eat any red or processed meat [48]. This estimate is consistent with the results of our meta-analysis.

Although we cannot rule out residual confounding, most studies included in the meta-analyses adjusted results by smoking, alcohol consumption, BMI and physical activity [18], [22]–[24], [26], [27], [50], [51], [54], [56], [57], [64] in addition to age, sex and energy; in several cohort studies the multivariate adjusted models also included folate intake [18], [24], [34], [50], and other studies additionally adjusted for aspirin or other anti-inflammatory drug use [23], [25], [53], [54]. Several potential confounders were not included in the final statistical models in some studies because, as the authors reported, their inclusion in the model did not substantially modified the relative risk estimates [19], [33], [49], [52], [60], [63].

### Implications

The remaining question is whether there is substantial potential for primary prevention of colorectal cancer through limiting the intake of red meat and processed meat in high meat consumers.

At a population level, the preventability estimates for red meat intake and colorectal cancer were 5% in the US, and the UK; and 7% in Brazil, and China; where 26%, 25%, 45% and 37% of the respective populations were estimated to consume more than 80 g of red meat per day [69]. Dietary and lifestyle factors are usually interrelated and it is likely that a change in a habit that is considered detrimental, such as high intake of red meat, will be accompanied by other healthful changes.

In the large prospective cohort of American Nurses (NHS), it was estimated that women who consumed high amounts of red and processed meat, did not exercise, had a low folate intake, and had a consistently excess in body weight experienced over 3.5 times' the cumulative incidence of colon cancer, by age 70 years, than women who maintained a low-risk lifestyle and diet (defined as consuming low amounts of red and processed meat, exercising regularly, consuming 400 µg/day of folate, and maintaining a low relative body weight) [48]. Under different scenarios for red meat consumption, reduction of physical inactivity, obesity, alcohol consumption, early adulthood cigarette smoking, and low intake of folic acid from supplements, the population attributable risk of colon cancer for the combined modifiable risk factors ranged from 39% to 55% of cancers in an American cohort of middle age men [70]. The preventability of colorectal cancer in United Kingdom through reduced consumption of red meat, increased fruit and vegetables, increased physical activity, limited alcohol consumption and weight control was estimated to be 31.5% of colorectal cancer in men and 18.4% in women [15], [71]. The preventability estimates of colorectal cancer through increasing intake of foods containing fiber, reducing intake of red and processed meat, alcohol, physical inactivity and body fatness were estimated to be close to 40% in USA, UK and Brazil, and 17% in China [69]. Measurement error might have attenuated the relative risk estimates in the individual studies in which the estimates are based, as well as in our meta-analyses.

### Conclusions

The current evidence from prospective studies supports limiting the amount of red and processed meat in the high consumers for colorectal cancer prevention. Primary prevention of colorectal cancer should emphasize modification of multiple diet and lifestyle factors.

## Supporting Information

Table S1 **Main characteristics of the prospective studies included in the dose-response meta-analyses.**
(DOC)

Table S2 **Studies or results not included in the dose-response meta-analyses and reasons for exclusion.**
(DOC)

Text S1 **Search strategy for the WCRF/AICR Continuous Update Project.**
(DOC)
