# Supplementary material for: Red and Processed Meat and Colorectal Cancer Incidence: Meta-Analysis of Prospective Studies
Source: PLoS One. 2011 Jun 6;6(6):e20456. doi: 10.1371/journal.pone.0020456 (PMC3108955; doi:10.1371/journal.pone.0020456)
Supplement: Table S1 — Main characteristics of the prospective studies included in the dose-response meta-analyses. (DOC) [file pone.0020456.s001.doc]

Table S1. Main characteristics of the prospective studies included in the dose-response meta-analyses.*

| **Author, year, study** | **Study characteristics** | **Exposure details** | **Subgroup** | **Total no. of cases in analysis** | **Exposure categories**  **/increment** | **RR (95% CI)**  **(Highest vs. lowest)** | **Adjustments** |
| --- | --- | --- | --- | --- | --- | --- | --- |
| Willett et al., 1990  Nurses’ Health Study, United States [58] | 88751 women  512488 person-years  34-59 years  61-item semi-quantitative FFQ | Red meat and processed meat (beef, pork or lamb as a main dish, as a sandwich, as a mixed dish, hamburger, hot dogs, preserved meats and bacon) | Female | 150 CC | g/day  ≥134 vs. ≤58 | 1.77 (1.09 - 2.88) | Age, total energy intake |
| Bostick et al., 1994  Iowa Women’s Health Study, United States [52] | 35216 women  167447 person-years  55-69 years  127-item semi-quantitative FFQ | Red meat  Beef, pork or lamb  Processed meat | Female | 212 CC | Servings/week  ≥11 vs. <4  >3 vs. <1  ≥3 vs. 0 | 1.04 (0.62 - 1.76)  1.21 (0.75 - 1.96)  1.51 (0.72 - 3.17) | Age, height, total energy intake, total vitamin E intake, total vitamin E intake by age, vitamin A supplement, parity; cofounders retained in final model were those tested at 0.1 level of significance |
| Giovannucci et al., 1994  Health Professional Follow-up Study, United States [55] | 47949 men  262710 person-years  6 years of follow-up  40-75 years  131-item semi-quantitative FFQ | Red meat and processed meat (beef, pork or lamb as a main dish, as a sandwich or mixed dish, hamburger, hot dogs, preserved meat and bacon) | Male | 201 CC | Median g/day  129.5 vs. 18.5 | 1.71(1.15 - 2.55) | Age, total energy |
| Chen et al., 1998  Physicians’ Health Study, United States [51]  (Nested case-control study) | 425 men  208 cases; 217 controls  40-84 years  FFQ | Red meat and processed meat (beef, pork or lamb as a main/mixed dish or sandwich, and hotdogs) | Male | 208 CRC | Servings/day  ≥1.1 vs. 0-0.5 | 1.17 (0.68 - 2.02) | BMI, alcohol intake, physical activity, matched by age and smoking |
| Singh and Fraser, 1998  California Seventh-day Adventists Study, United States [53] | 32051 men and women  178544 person-years  At least 25 years of age  FFQ | Red meat (beef or pork) |  | 127 CC | Times/week  ≥1 vs. 0 | 1.41 (0.90 - 2.21) | Age, sex, BMI, smoking habits, alcohol intake, physical activity, family history of cancer, aspirin use |
| Pietinen et al., 1999  Alpha-Tocopherol, Beta-Carotene Cancer (ATBC) Prevention Study, Finland [57] | 27111 male smokers  8 years follow-up  50-69 years  Dietary history questionnaire | Red meat and processed meat  Red meat (Beef, pork or lamb)  Processed meat | Male | 185 CRC | Median g/day  203 vs. 79  99 vs. 35  122 vs. 26 | 1.1 (0.7 - 1.7)  0.8 (0.5 - 1.2)  1.2 (0.7 - 1.8) | Age, BMI, smoking years, energy intake, alcohol intake, physical activity, calcium intake, education level, supplement group |
| Jarvinen et al., 2001  Finnish Mobile Clinic Health Examination Survey study, Finland [61] | 9959 men and women  Maximum 32 years of follow-up  Mean age 39 years  Questionnaire | Red meat | (Overall) | 109 CRC  63 CC  46 RC | g/day  Men  >206 vs. <94  Women  134 vs. <61 | 1.50 (0.77 - 2.94)  1.34 (0.57 - 3.15)  1.82 (0.60 - 5.52) | Age, sex, BMI, smoking, total energy intake, vegetable, fruits and cereal intake, occupational group, geographical area |
| Tiemersma et al., 2002  Dutch Prospective Monitoring Project on Cardiovascular Disease Risk Factors, The Netherlands [63]  (Nested case-control study) | 639 men and women  102 cases  537 controls  20-59 years  FFQ | Fresh red meat |  | 102 CRC | Times/week  ≥5 vs. 0-3 | 1.6 (0.9 - 2.9) | Age, sex, height, alcohol intake, total energy intake, study centre, other covariates tested but no material change to risk estimates |
| Flood et al., 2003  Breast Cancer Detection Demonstration Project (BCDDP) follow-up cohort, United States [33] | 45496 women  386716 person-years  8.5 years follow-up  Multiethnic  Mean age 62 years  62-item National Cancer Institute/Block FFQ | Red and processed meat (bacon, beef, hamburger, ham or other lunch meat, hotdogs, liver, pork, sausage and meat from mixed dishes)  Processed meat (bacon, ham or other lunch meat, hotdogs and sausage) | Female | 311 CRC | Median g/1000kcal/day  52.2 vs. 6.1  22.2 vs. 0.02 | 1.10 (0.83 - 1.45)  1.00 (0.76 - 1.31) | Total energy, other covariates tested but no material change to risk estimates |
| English et al., 2004  Melbourne Collaborative Cohort Study, Australia [60] | 37112 men and women  9 years of follow-up  27-75 years  121-item FFQ | Red meat (veal or beef schnitzel, steak, balls or meatloaf, mixed dishes with beef, lamb or pork, rabbit or other game)  Processed meat (salami, sausages, bacon, ham, corned beef or luncheon meats) |  | 452 CRC  283 CC  169 RC  452 CRC  283 CC  169 RC | 1 time/week | 1.03 (0.98 - 1.08)  1.00 (0.94 - 1.07)  1.08 (0.99 - 1.16)  1.07 (1.02 - 1.13)  1.07 (1.00 - 1.14)  1.08 (0.99 - 1.18) | Age, sex, country of birth, total energy intake, fat intake, cereal product intake, other covariates tested but no material change to risk estimates |
| Lin et al., 2004  Women's Health Study, United States [56] | 37547 women  8.7 years follow-up  At least 45 years old  131-item FFQ | Red meat and processed meat (red meat - beef or lamb as main dish; beef, pork or lamb in a sandwich; hotdogs, bacon, processed meats and hamburgers)  Processed meat (hotdogs, processed meats and bacon) | Female | 202 CRC | Median servings/day  1.42 vs. 0.13  0.5 vs. 0 | 0.66 (0.40 - 1.09)  0.85 (0.53 - 1.35) | Age, BMI, smoking habits, alcohol intake, physical activity, total energy intake, family history of colorectal cancer, history of polyps, postmenopausal hormone use, randomized treatment assignment |
| Wei et al., 2004  Health Professionals Follow-up Study, United States [18] | 46632 men  14 years follow-up  602 CRC cases  40-75 years  131-item semi-quantitative FFQ | Beef, pork or lamb  Processed meat | Male | 467 CC  135 RC | ≥5 servings/week vs. 0-3 servings/month  ≥5 serving/week vs. 0 serving/month | 1.35 (0.80 - 2.27)  0.90 (0.34 - 2.45)  1.27 (0.87 - 1.85)  1.06 (0.48 - 2.33) | Age, height, BMI, pack-years of smoking before age 30, energy intake, alcohol intake, physical activity, family history, history of endoscopy, calcium and folate intake, processed meat, beef, pork or lamb as a main dish |
| Wei et al., 2004  Nurses’ Health Study, United States [18] | 87733 women  24 years follow-up  876 CRC cases  30-55 years  61-item semi-quantitative FFQ | Beef, pork or lamb  Processed meat | Female | 670 CC  203 RC  668 CC  202 RC | ≥5 servings/week vs. 0-3 servings/month | 1.31 (0.73 - 2.36)  0.92 (0.31 - 2.71)  1.32 (0.95 - 1.83)  0.73 (0.33 - 1.59) | Age, height, BMI, pack-years of smoking before age 30, energy intake, alcohol intake, physical activity, family history, history of endoscopy, calcium and folate intake, processed meat, beef, pork or lamb as a main dish |
| Brink et al., 2005  The Netherlands Cohort Study [49], The Netherlands  (Case Cohort Study) | 2948 men and women  608 CRC cases  14738 person-years  7.3 years follow-up  55-69 years  150-item semi-quantitative FFQ | Preserved meat (meat product, cured, smoked or fermented meat) |  | 448 CC  160 RC | 15g/day | 1.05 (0.94 - 1.16)  0.97 (0.84 - 1.13) | Age, sex, BMI, smoking habits, total energy intake, family history of colorectal cancer; only factors found to contribute substantially to the multi-variate model were included as covariates |
| Chao et al., 2005  Cancer Prevention Study (CPS) II Nutrition Cohort, United States [54] | 148610 men and women  1667 CRC cases  9 years follow-up  Multiethnic  50-74 years  68-item modified Block FFQ | Red meat and processed meat (bacon, sausage, hamburgers or ground beef dish, beef, liver, pork, hotdogs, ham)  Processed meat (bacon, sausage, hotdogs and ham or lunchmeat) | Male  Female  (male and female) | 665 CC  532 CC  667 PC  408 DC  470 RC | g/week  ≥801 vs. ≤180  ≥561 vs. ≤90  g/week  ≥241 vs. 0  ≥121 vs. 0 | 1.30 (0.93 - 1.81)  0.98 (0.68 - 1.40)  1.27 (0.91 - 1.76)  0.71 (0.47 - 1.07)  1.71(1.15 - 2.52)  1.11 (0.80 - 1.54)  1.16 (0.85 - 1.57)  0.97 (0.72 - 1.29)  1.39 (0.94 - 2.05)  1.26 (0.86 - 1.83) | Age, (sex), BMI, smoking habits, intake of wine, beer or liquor, recreational physical activity, total energy, fruit, vegetable and high-fiber grain foods intakes, education, multivitamin use, aspirin use, use of hormone therapy (women) |

| Larsson et al.,  2005  Swedish Mammography Cohort, Sweden [34] | 61433 women  855585 person-years  40-75 years  67-item FFQ | Red meat and processed meat (whole beef, chopped meat, minced meat, bacon, hot dogs, ham or other lunch meat, blood pudding, kidney or liver and pate)  Beef, pork or lamb  Processed meat (bacon, hot dogs, ham or other lunch meat and blood pudding) | Female | 733 CRC  234 PC  155 DC  230 RC | 100 g/day  114 vs.37 g/day  100 g/day  114 vs.37 g/day  Servings/week  5.5 vs. 1.5  g/day  41 vs. 6 | 1.20 (0.99 - 1.45)  1.03 (0.67 - 1.60)  1.70 (1.31 - 2.21)  1.28(0.83 - 1.98)  1.22 (0.98 - 1.53)  1.10 (0.74 - 1.64)  1.99 (1.26 - 3.14)  1.08 (0.72 - 1.62)  1.07 (0.85 - 1.33)  1.02 (0.69 - 1.52)  1.39 (0.86 - 2.24)  0.90 (0.60 - 1.34) | Age, BMI, alcohol intake, total energy intake, saturated fat, calcium, folate, fruit, vegetables, whole-grain foods, educational level, fish and poultry intake (for red and processed meat and beef, pork or lamb) |
| --- | --- | --- | --- | --- | --- | --- | --- |
| Norat et al., 2005  European Prospective Investigation into Cancer and Nutrition (EPIC) [50] | 478040 men and women  2279075 person-years  10 European countries  21-83 years  Country-specific 88-266-item FFQ | Red meat and processed meat (fresh, minced and frozen beef, veal, pork and lamb (red meat) plus pork and beef preserved by methods other than freezing  Red meat included all fresh, minced, and frozen beef, veal, pork, and lamb.  Processed meat (mostly pork and beef preserved by methods other than freezing) |  | 1329 CRC  855 CC  474 RC | 100g/day | 1.25 (1.09 - 1.41)  1.26 (1.07 - 1.48)  1.22 (0.99 - 1.51)  1.21 (1.02 - 1.43)  1.20 (0.96 - 1.48)  1.23 (0.94 - 1.62)  1.32 (1.07 - 1.63)  1.39 (1.06 - 1.82)  1.22 (0.87 - 1.71) | Age, sex, height, weight, alcohol intake, smoking status, occupational physical activity, energy from nonfat sources except alcohol, energy from fat sources, fibre intake, stratification by study centre |
| Balder et al., 2006  The Netherlands Cohort Study, The Netherlands  (Case Cohort Study) [64] | 1535 cases; 4371 non-cases from 120852 men and women  9.3 years follow-up  55-69 years  150-item semi-quantitative FFQ | Processed meat | Male  Female | 869 CRC  666 CRC | ≥20 vs. 0 g/day | 1.18 (0.84 - 1.64)  1.05 (0.74 - 1.48) | Age, BMI, smoking status, alcohol intake, non-occupational activity, total energy intake, vegetable intake, family history of colorectal cancer |
| Berndt et al., 2006  CLUE II Cohort, United States [19]  (Case Cohort Study) | 202 cases; 1583 subcohort of men and women  13.5 years follow-up  Mean age 48 years  FFQ | Red meat included hamburgers, cheeseburgers, meatloaf, beef, beef stew, pork, hot dogs, bacon, sausage, ham, bologna, salami, and other lunch meats |  | 202 CRC | g/day  ≥86.3 vs. <44.0 | 1.32 (0.86 - 2.02) | Age, race and energy, adjustment for sex, BMI and education did not significantly alter the results and were not included in the model |
| Oba et al., 2006  Takayama City Cohort, Japan [27] | 30221 men and women  213 CRC cases  8 years  35-101 years  169-item semi-quantitative FFQ | Red meat (beef and pork)  Processed meat, ham sausage bacon and Chinese style roast pork | Male  Female | 111 CC  102 CC | Median g/day  56.6 vs. 18.7  42.3 vs. 10.7  20.3 vs. 3.9  16.3 vs. 3 | 1.03 (0.64 - 1.66)  0.79 (0.49 - 1.28)  1.98 (1.24 - 3.16)  0.85 (0.50 - 1.43) | Age, height, BMI, pack-years of smoking, alcohol intake, physical activity, total energy intake |
| Cross et al., 2007  National Institute of Health- American Association of Retired Persons (NIH-AARP) Diet and Health Study, United States [22] | 494036 men and women  6.8 years follow-up  Multiethnic  50-71 years  124-item FFQ | Red meat and processed meat (all types of beef, pork and lamb, included bacon, beef, cold cuts, ham hamburger, hotdogs, liver, pork, sausage and steak)  Processed meat (bacon, red meat sausage, poultry sausage, luncheon meats/cold cuts (red/white meat), ham, regular/low fat hotdogs) |  | 5107 CRC | Median g/1000kcal/day  62.7 vs. 9.8  22.6 vs. 1.6 | 1.24 (1.12 - 1.36)  1.20 (1.09 - 1.32) | Age, sex, ethnicity, BMI, smoking habits, alcohol intake, physical activity, total energy intake, fruit and vegetable intake, education level, marital status, family history of cancer |

| Kabat et al., 2007  Canadian National Breast Screening Study, Canada (NBSS) [24] | 48666 women  617 cases  16.4 years of follow-up  Canada  40-59 years  86-item FFQ | Red meat  (beef, pork, ham, bacon, pork-based luncheon meats and veal) | Female | 617 CRC  428 CC  195 RC | g/day  ≥40.30 vs. ≤14.24 | 1.12 (0.86 - 1.46)  0.88 (0.64 - 1.21)  1.95 (1.21 - 3.16) | Age, BMI, pack-years of smoking, alcohol intake, physical activity, total energy intake, fat and fibre intake, dietary folic acid, educational level, menopausal status, oral contraceptive use, hormone replacement therapy use |
| --- | --- | --- | --- | --- | --- | --- | --- |
| Lee et al., 2009  Shanghai Women's Health Study, China [25] | 73224 women  540156 person-years  7.4 years follow-up  40-70 years  Quantitative FFQ | Red meat | Female | 394 CRC  236 CC  158 RC | g/day  ≥67 vs. <24 | 0.8 (0.6 - 1.1)  0.9 (0.6 - 1.5)  0.6 (0.3 - 1.1) | Age, total energy intake, fibre intake, tea consumption, education level, income, non-steroidal anti-inflammatory drugs use, survey season |
| Nothlings et al., 2009  Multiethnic Cohort Study, United States [26]  (Nested case-control study) | 2531 men and women  1009 cases  1522 controls  Multiethnic  67-75 years  >180-item quantitative FFQ | Red meat  Processed meat |  | 1009 CRC | g/1000kcal/day  ≥26.0 vs. <10.4  ≥11.0 vs. <3.5 | 0.96 (0.74 - 1.23)  1.08 (0.83 - 1.39) | Age, sex, ethnicity, BMI, smoking status, pack-years of smoking, alcohol intake, physical activity, intake of dietary fibre, calcium, Vitamin D and folic acid, family history of colorectal cancer |
| Cross et al., 2010  National Institute of Health- American Association of Retired Persons (NIH-AARP) Diet and Health Study, United States [21] | 300948 men and women  2719 CRC cases  7.2 years follow-up  Multiethnic  50-71 years  124-item FFQ | Red meat (all types of beef, pork, and lamb, including bacon, beef, cold cuts, ham, hamburger, hotdogs, liver, pork, sausage, steak, and meat added to complex food mixtures)  Processed meat (included bacon, red meat sausage, poultry sausage, luncheon meats/cold cuts (red and white meat), ham, regular hotdogs, low-fat hotdogs made from poultry, and meat added to complex food mixtures) |  | 1995 CC  724 RC | 100g/day | 1.20 (1.05 - 1.36)  1.31 (1.07 - 1.61)  1.13 (0.88 - 1.45)  1.38 (0.93 - 2.05) | Person-years, gender, BMI, smoking, intake of total energy, fiber, and dietary calcium, white meat, education  As for the analysis on red meat but with non-processed meat instead of white meat  The final multivariate models only contained variables that changed the risk estimates by ≥10% or were established risk factors for colorectal cancer |
| Fung et al., 2010  Health Professional Follow-up Study, United States [23] | 45490 men  1032 CRC cases  ≤26 years follow-up  40-75 years  131-item semi-quantitative FFQ | Red or processed meat |  | 1032 CRC | 1 serving/day | 1.08 (0.97-1.21) | Age, BMI, pack-years of smoking, alcohol intake, physical activity, energy intake, multivitamin use, aspirin use, family history, colonoscopy, history of polyps |
| Fung et al., 2010  Nurses’ Health Study, United States [23] | 87256 women  1432 CRC cases  ≤20 years follow-up  30-55 years  61-item semi-quantitative FFQ | Red or processed meat |  | 1432 CRC | 1 serving/day | 1.12 (0.99-1.26) | Age, BMI, pack-years of smoking, alcohol intake, physical activity, energy intake, multivitamin use, aspirin use, family history, colonoscopy, history of polyps |

*CRC – colorectal cancer; CC – colon cancer; PC – proximal colon cancer; DC – distal colon cancer; RC – rectal cancer; RR – relative risk; CI – confidence interval; BMI – body mass index; FFQ – food frequency questionnaire
