# Supplementary material for: Red and Processed Meat and Colorectal Cancer Incidence: Meta-Analysis of Prospective Studies
Source: PLoS One. 2011 Jun 6;6(6):e20456. doi: 10.1371/journal.pone.0020456 (PMC3108955; doi:10.1371/journal.pone.0020456)
Supplement: Table S2 — Studies or results not included in the dose-response meta-analyses and reasons for exclusion. (DOC) [file pone.0020456.s002.doc]

Table S2. Studies or results not included in the dose-response meta-analyses and reasons for exclusion.*

| **Author, year, study** | **Study characteristics** | **Exposure details** | **Subgroup** | **Total no. of cases in analysis** | **Exposure categories**  **/increment** | **RR (95% CI)** | **Adjustments** | **Reasons to exclude in the dose-response meta-analysis/remarks** |
| --- | --- | --- | --- | --- | --- | --- | --- | --- |
| Willett et al., 1990  Nurses’ Health Study, United States [58] | 88751 women  512488 person-years  34-59 years  61-item semi-quantitative FFQ | Red meat (beef, pork or lamb)  Processed meat | Female | 150 CC | <1 time/month  <1 times/week  2-4  5-6  ≥7  <1 time/month  1-3  1 times/week  2-4  >=5 | 1.00  1.39 (0.75-2.56)  1.50 (0.84-2.70)  1.84 (0.90-3.75)  2.49 (1.24-5.03)  P trend = 0.01  1.00  1.09 (0.70-1.69)  1.45 (0.91-2.31)  1.86 (1.16-2.98)  1.21 (0.53-2.72)  P trend = 0.04 | Age, total energy intake | Superseded by Wei et al., 2004 (results on red and processed meat from article was included in the meta-analysis) |
| Giovannucci et al., 1994  Health Professional Follow-up Study, United States [55] | 47949 men  262710 person-years  6 years of follow-up  40-75 years  131-item semi-quantitative FFQ | Red meat and processed meat (beef, pork or lamb as a main dish, as a sandwich or mixed dish; hamburger, hotdogs, preserved meat and bacon)  Red meat (beef, pork or lamb)  Processed meat | Male | 251 CRC  69 PC  89 DC  46 RC  201 CC  202 CC | 129.5 vs. 18.5 g/day  0 serving/month  1-3  1 serving/week  2-4  >=5 | 1.66 (1.14-2.42)  P trend = 0.003  0.87 (0.43-1.76)  P trend = 0.85  1.78 (0.97-3.25)  P trend = 0.07  1.22 (0.36-4.14)  1.00  1.92 (1.03-3.60)  1.69 (0.92-3.10)  2.11 (1.13-3.92)  3.57 (1.58-8.06)  P trend = 0.01  1.00  1.25 (0.87-1.80)  1.40 (0.92-2.13)  1.67 (1.06-2.61)  1.16 (0.44-3.04)  P trend = 0.06 | Age, total energy intake | Risk estimates for Q5 versus Q1 only; included in the higher versus lower meta-analysis (results on colon cancer from article was included in the meta-analysis)  Superseded by Wei et al., 2004 |
| Goldbohm et al., 1994  The Netherlands cohort study, The Netherlands [72]  (Case cohort study) | 120852 men and women  3.3 years of follow-up  55-69 years  150-item semi-quantitative FFQ | Processed meat  Beef  Pork  Sausages  Bacon  Ham |  | 215 CC | 0 g/day  0-10  10-20  >=20  15 g/day | 1  1.23 (0.76-1.98)  1.43 (0.87-2.35)  1.72 (1.03-2.87)  0.96 (0.87-1.06)  0.99 (0.92-1.06)  1.27 (1.04-1.55)  1.25 (0.84-1.88)  1.04 (0.78-1.39) | Age, sex, total energy intake, dietary fiber intake | Superseded by Brink et al., 2005  Specific meat exposures |
| Gaard et al., 1996  Norwegian National Health Screening Service Study, Norway [67] | 50535 men and women  145 cases  11.4 years follow-up  20-53 years  FFQ | Poached or fried sausages | Male  Female | 85 CC  60 CC | <1 times/month  1-2  3-4  >=5 | 1.00  1.52 (0.74-3.14)  1.46 (0.69-3.06)  1.98 (0.70-5.58)  P trend = 0.35  1.00  1.45 (0.63-3.35)  1.85 (0.79-4.31)  3.50 (1.02-11.90)  P trend = 0.03 | Age, attained age | Specific exposure on fried sausages |
| Kato et al., 1997  New York University Women Health's Study, United States [62] | 14272 women  105044 person-years  34-65 years  Semi-quantitative FFQ | Red meat  Processed meat (ham and sausages) | Female | 100 CRC | Q1  Q2  Q3  Q4 | 1.00  1.28 (0.72-2.28)  1.27 (0.71-2.28)  1.23 (0.68-2.22)  1.00  1.39 (0.81-2.38)  1.38 (0.79-2.42)  1.09 (0.59-2.02) | Age, total energy intake, education level, place of enrolment | Exposure levels not quantified (included in the higher versus lower meta-analysis) |
| Sellers et al., 1998  Iowa Women's Health Study, United States [44] | 35216 women  241 cases  10 years of follow-up  55-69 years  127-semi-quantitative FFQ | Red meat, includes liver, hamburger, beef stew, beef and venison  Preserved meat (  Nitrate meat includes bacon, hotdogs, and processed meats) | No family history of colon cancer  Family history of colon cancer  No family history of colon cancer  Family history of colon cancer | 180 CRC  61 CRC  180 CRC  61 CRC | ≤3.5 servings/week  3.6-7.0  >=7.1  <=0.5 servings/week  0.6-1.5  >=1.6 | 1.0  1.2 (0.8-1.8)  1.3 (0.8-2.0)  1.0  0.9 (0.5-1.7)  1.0 (0.5-2.1)  1.0  1.0 (0.7-1.4)  1.0 (0.7-1.4)  1.0  1.3 (0.7-2.3)  0.8 (0.4-1.6) | Age, total energy intake, history of rectal colon polyps | Subgroup by family history of colon cancer; number of non-cases per quantile level was not provided; selected Bostick et al., 1994 |
| Fraser et al., 1999  California Seventh-day Adventists study, United States [45] | 34198 men and women  6 years  25-100 years  FFQ | Red meat | Infrequent use of legumes  <1 time/week white meat intake | 112 CC | Yes vs. no  never  < 1 time/wk  >=1 time/wk | 2.68 (1.24-5.78)  1.00  1.37 (0.85-2.20)  1.86 (1.15-3.02)  P trend = 0.01 | Unadjusted | Subgroup by dietary habits, selected Singh and Fraser, 1998 |
| Knekt et al., 1999  Finnish Mobile Clinic Health Examination Survey study, Finland [66] | 9985 men and women  24 years of follow-up  Diet history | Processed meat (cured meat and meat products) |  | 73 CRC | Q1  Q2  Q3  Q4 | 1.00  1.48 (0.77-2.84)  1.28 (0.63-2.57)  1.84 (0.98-3.47) | Age, sex, smoking, total energy intake, municipality | Exposure levels not quantified (included in the higher versus lower meta-analysis) |
| Ma et al., 2001  Physicians' Health Study, United States  (Nested Case-Control study) [46] | 511 men  193 cases  318 controls  40-84 years  19-item FFQ | Red meat and processed meat (beef as a main dish, as a sandwich and hotdogs) | Male | 193 CRC | 0-0.5 servings/day  0.6-0.9  0.9-2.1 | 1  1.12 (0.68-1.83)  0.98 (0.60-1.60) | Age, BMI, smoking habits, alcohol intake, physical activity, supplement and aspirin use, molar ratio of insulin-like growth factor (IGF)-I to IGF binding protein-3 | Selected Chen et al., 1998 that has more number of cases. |

| Tiemersma et al., 2002  Dutch Prospective Monitoring Project on Cardiovascular Disease Risk Factors, The Netherlands [63]  (Nested case-control study) | 639 men and women from a cohort of 36000 over 8.5 years  102 cases  537 controls  20-59 years  FFQ | Sandwiches with meat filling  Sausages as snacks |  | 102 CRC | 0-1 times/day  >=2  Yes vs. no | 1.0  0.7 (0.4-1.1)  0.9 (0.6-1.3) | Age, sex, height, alcohol intake, total energy intake, study centre, other covariates tested but no material change to risk estimates | Specific meat exposures (results on red meat from article was included in the meta-analysis) |
| --- | --- | --- | --- | --- | --- | --- | --- | --- |
| Chen et al., 2003  Chinese Jiashan screening study, China [68]  (Nested case-control study) | 196 cases (84 colon, 112 rectal), 980 controls from 64693 men and women  10 years follow-up  30->80 years  Questionnaire, interviewed | Pork |  | 84 CC | Yes vs. no | 1.481 (0.846-2.593) | Unadjusted, matched by age, sex, country/town | Specific meat exposure |
| Wu et al., 2004  Health Professional Follow-up Study, United States [47] | 47311 men  Max 14 years follow-up  561 CC cases  40-75 years  131-item FFQ | Total red meat  Beef, pork or lamb as main dish  Processed meat | Male | Not provided | highest vs. lowest | 1.40 (0.92-2.13)  1.42 (0.86-2.35)  1.68 (1.21-2.33) | Multivariate adjusted | Highest versus lowest comparison only.  Results provided in text of article; insufficient to include in meta-analysis |
| Brink et al., 2005  The Netherlands Cohort Study, The Netherlands  (Case Cohort Study) [49] | 2948 men and women  14738 person-years  7.3 years follow-up  608 cases  55-69 years  150-item semi-quantitative FFQ | Beef  Pork  Sausages, meat other than fresh meat |  | 448 CC  160 RC | 22.5g/day  30.3g/day  6.4g/day | 1.06 (0.97-1.17)  0.94 (0.78-1.14)  0.93 (0.83-1.04)  0.87 (0.72-1.03)  1.08 (0.98-1.18)  1.00 (0.88-1.15) | Age, sex, BMI, smoking habits, total energy intake, family history of colorectal cancer; only factors found to contribute substantially to the multi-variate model were included as covariates | Specific meat exposures  (results on processed meat from article was included in the meta-analysis) |
| Chan et al., 2005  Nurses’ Health Study, United States [59]  (Nested case-control study) | 626 women from a cohort of 32826  183 cases  443 controls  Mean age 60.6 years  61-item semi-quantitative FFQ | Red meat (beef, pork and lamb as main dish) | Female | 183 CRC | > 0.5 vs. <=0.5 serving/day | 1.21 (0.85-1.72) | Age, BMI, smoking history, multivitamin use, aspirin use, family history of colorectal cancer, postmenopausal hormone use, history of endoscopy matched by year of birth and month/year of blood collection | Two exposure categories only; same study as Willett et al.’s and Wei et al.’s but results were on colorectal cancer (included in the higher versus lower meta-analysis) |
| Larsson et al.,  2005  Swedish Mammography Cohort, Sweden [34] | 61433 women  855585 person-years  733 CRC cases  40-75 years  67-item FFQ | Red meat (beef, pork or lamb) | Female | 234 PC  155 DC | <2 servings/week  2-3  3-4  >=4 | 1.00  0.90 (0.65-1.24)  0.78 (0.45-1.17)  1.10 (0.74-1.64)  P trend = 0.90  1.00  1.26 (0.84-1.90)  0.98 (0.55-1.75)  1.99 (1.26-3.14)  P trend = 0.01 | Age, BMI, alcohol intake, total energy intake, saturated fat, calcium, folate, fruit, vegetables, whole-grain foods, fish and poultry intake, educational level | Outcome was proximal and distal colon cancer; meta-analysis was not performed as this is the only study provided sufficient information (results on colorectal and rectal cancer from article were included in the meta-analyses) |

| Luchtenborg et al., 2005  The Netherlands Cohort Study, the Netherlands [41]  (Case cohort study) | 2948 men and women  588 CRC cases  14272 person-years  7.3 years follow-up  The Netherlands  55-69 years  150-item semi-quantitative FFQ | Beef  Pork  Processed meat (preserved meat)  Sausages, meat other than fresh meat |  | 434 CC  154 RC | 22.4 g/day  30.3 g/day  15g/day  Users vs. non-users | 1.06 (0.97-1.17)  0.94 (0.78-1.14)  0.93 (0.84-1.04)  0.87 (0.73-1.03)  1.05 (0.94-1.17)  0.97 (0.83-1.14)  1.16 (0.92-1.45)  1.34 (0.94-1.90) | Age, sex, BMI, smoking status, total energy intake, family history of colorectal cancer | Specific meat exposures; superseded by Brink et al., 2005 |
| --- | --- | --- | --- | --- | --- | --- | --- | --- |
| Norat et al., 2005  European Prospective Investigation into Cancer and Nutrition (EPIC) [50] | 478040 men and women  2279075 person-years  1329 CRC cases  10 European countries  21-83 years  Country-specific 88-266-item FFQ | Red meat and processed meat (fresh, minced and frozen beef, veal, pork and lamb plus pork and beef preserved by methods other than freezing)  Red meat included all fresh, minced, and frozen beef, veal, pork, and lamb.  Processed meat (mostly pork and beef preserved by methods other than freezing)  Beef or veal  Pork  Lamb  Other processed meat/sausages  Bacon  Ham |  | 351 Right colon cancer  391 Left colon cancer  1329 CRC | <=10 g/day  20-40  40-80  80-160  >=160  <10 g/day  10-20  20-40  40-80  >=80  <10 g/day  10-20  20-40  40-80  >=80  Q5 vs. Q1 | 1.00  0.89 (0.55-1.45)  0.99 (0.64-1.51)  1.09 (0.70-1.72)  1.03 (0.56-1.91)  1.00  1.34 (0.75-2.39)  1.34 (0.79-2.29)  1.55 (0.89-2.69)  1.51 (0.76-3.02)  1.00  1.13 (0.70-1.84)  1.00 (0.65-1.54)  1.36 (0.90-2.07)  1.18 (0.73-1.91)  1.00  1.07 (0.68-1.68)  1.10 (0.65-1.63)  1.11 (0.75-1.64)  1.24 (0.80-1.94)  1.00  1.04 (0.73-1.49)  0.95 (0.67-1.34)  1.17 (0.80-1.70)  1.19 (0.70-2.01)  1.00  1.30 (0.92-1.83)  1.32 (0.94-1.85)  1.45 (1.00-2.11)  1.48 (0.87-2.53)  1.03 (0.86-1.24)  1.18 (0.95-1.48)  1.22 (0.96-1.55)  1.05 (0.84-1.32)  0.96 (0.79-1.17)  1.12 (0.90-1.37) | Age, sex, height, weight, alcohol intake, smoking status, occupational physical activity, energy from nonfat sources except alcohol, energy from fat sources, fibre intake, stratification by study centre | Insufficient information to be included in the dose-response meta-analysis; included in the higher versus lower meta-analysis (results on colorectal, colon and rectal cancer from article were included in the meta-analyses)  Specific meat exposures |

| Sato et al., 2006  Miyagi Cohort Study, Japan [28] | 41835 men and women  474 cases  11 years of follow-up  40-64 years  40-item FFQ | Beef  Pork (excluding ham and sausages)  Ham and sausages |  | 368 CRC  217 CC  115 PC  75 DC  155 RC  396 CRC  240 CC  123 PC  85 DC  159 RC  358 CRC  217 CC  113 PC  76 DC  144 RC | 0 g/day  3.5  7.4  0 g/day  1.8  7.5  26.3  0 g/day  1.1  4.5  15.8 | 1.00  1.30 (1.04-1.62)  0.93 (0.67-1.30)  P trend = 0.63  1.00  1.33 (1.00-1.78)  0.84 (0.54-1.32)  P trend = 0.96  1.00  1.03 (0.69-1.54)  0.97 (0.55-1.70)  P trend = 0.93  1.00  2.40 (1.40-4.10)  1.06 (0.46-2.43)  P trend = 0.31  1.00  1.23 (0.87-1.73)  1.01 (0.62-1.67)  P trend = 0.58  1.00  1.02 (0.68-1.55)  1.13 (0.77-1.68)  1.13 (0.79-1.74)  P trend = 0.31  1.00  1.19 0.68-2.11)  1.34 (0.78-2.29)  1.46 (0.81-2.62)  P trend = 0.15  1.00  0.97 (0.47-1.99)  0.99 (0.51-1.95)  1.05 (0.50-2.22)  P trend = 0.80  1.00  1.47 (0.50-4.3)  1.88 (0.67-5.25)  1.90 (0.63-5.74)  P trend = 0.18  1.00  0.81 (0.44-1.47)  0.85 (0.49-1.48)  0.74 (0.39-1.42)  P trend = 0.72  1.00  0.98 (0.74-1.31)  1.02 (0.77-1.36)  0.91 (0.61-1.35)  P trend = 0.99  1.00  1.00 (0.70-1.42)  0.86 (0.60-1.25)  0.75 (0.45-1.27)  P trend = 0.25  1.00  1.28 (0.78-2.11)  0.86 (0.50-1.46)  0.69 (0.32-1.51)  P trend = 0.20  1.00  0.66 (0.36-1.20)  0.79 (0.44-1.41)  0.65 (0.28-1.55)  P trend = 0.50  1.00  0.91 (0.56-1.47)  1.21 (0.77-1.91)  1.10 (0.60-2.03)  P trend = 0.29 | Age, sex, BMI, smoking status, alcohol intake, walking, total energy intake, fat, calcium and fibre intake, educational level, family history of cancer | Specific meat exposures  Number of non-cases per category level was not provided (included in the higher versus lower meta-analysis) |
| --- | --- | --- | --- | --- | --- | --- | --- | --- |

| Cross et al., 2007  National Institute of Health- American Association of Retired Persons (NIH-AARP) Diet and Health Study, United States [22] | 494036 men and women  5107 CRC cases  6.8 years follow-up  Multiethnic  50-71 years  124-item FFQ | Red meat and processed meat (all types of beef, pork and lamb, included bacon, beef, cold cuts, ham hamburger, hotdogs, liver, pork, sausage and steak)  Processed meat (bacon, red meat sausage, poultry sausage, luncheon meats/cold cuts (red/white meat), ham, regular/low fat hotdogs) |  | 3689 CC  1418 RC | Q5 vs. Q1 | 1.17 (1.05-1.31)  1.45 (1.20-1.75)  1.18 (1.06-1.32)  1.24 (1.03-1.49) | Age, sex, ethnicity, BMI, smoking habits, alcohol intake, physical activity, total energy intake, fruit and vegetable intake, education level, marital status, family history of cancer | Presented risk estimates for Q5 compared to Q1 only (results on colorectal cancer were included in the meta-analyses) |
| --- | --- | --- | --- | --- | --- | --- | --- | --- |
| Butler et al., 2008  Singapore Chinese Health study, Singapore [20] | 61321 Chinese men and women  9.8 years  45-74 years  165-item FFQ | Red meat  Processed meat (preserved meat) |  | 961 CRC | Q4 vs. Q1 | - 1. (0.82-1.26)   1.16 (0.95-1.41) | Age, sex, BMI, smoking habits, alcohol intake, physical activity, total energy intake, dialect group, exposure assessment, diabetes, educational level, family history of colorectal cancer | Results for the highest versus lowest comparison only (included in the higher versus lower meta-analysis) |
| Sorensen et al., 2008  Diet, Cancer and Health Study, Denmark  (Case-cohort study) [43] | 57000 men and women  379 cases  769 controls  10 years follow-up  50-64 years  192-item FFQ | Red meat (beef, veal, pork, lamb, offal)  Processed meat (bacon, smoked ham, salami, frankfurter, cumberland sausage, cold cuts and liver pate and processed fish) |  | 379 CRC | 25g/day | - 1. (0.97-1.09)   0.99 (0.84-1.16) | BMI, smoking status, alcohol intake, intake of fiber, poultry and fish, hormone replacement therapy  BMI, smoking status, alcohol intake, fibre intake, hormone replacement therapy | Component study of EPIC, selected pooled results from Norat et al., 2005 |
| Wei et al., 2009  Nurses’ Health Study, United States [48] | 83767 women  1607643 person-years  30-55 years  61-item semi-quantitative FFQ | Processed meat plus red meat | Female | 701 CC | 1 serving/day | 1.20 (0.95-1.52) | Age, BMI, height, smoking habits, physical activity, folate intake, family history of colon cancer , aspirin use, postmenopausal hormone use, sigmoidoscopy/colonoscopy | Cumulative risk for age 30 – 70 years |

| Cross et al., 2010  National Institute of Health- American Association of Retired Persons (NIH-AARP) Diet and Health Study, United States [21] | 300948 men and women  7.2 years follow-up  Multiethnic  50-71 years  124-item FFQ | Red meat (all types of beef, pork, and lamb, including bacon, beef, cold cuts, ham, hamburger, hotdogs, liver, pork, sausage, steak, and meat added to complex food mixtures)  Processed meat (included bacon, red meat sausage, poultry sausage, luncheon meats/cold cuts (red and white meat), ham, regular hotdogs, low-fat hotdogs made from poultry, and meat added to complex food mixtures)  Unprocessed red meat |  | 2719 CRC  1150 PC  787 DC | 100 g/day  61.6 vs. 9.5 g/1000kcal  100g/day  22.3 vs. 1.6 g/1000kcal  Q5 vs Q1 | 1.23 (1.10-1.36)  1.15 (0.94-1.41)  P trend = 0.024  1.29 (1.00-1.66)  P trend = 0.018  1.19 (0.96-1.48)  1.09 (0.89-1.33)  P trend = 0.245  1.10 (0.86-1.41)  P trend = 0.363  1.13 (0.98-1.30) | Person-years, gender, BMI, smoking, intake of total energy, fiber, and dietary calcium, white meat, education  As for the analysis on red meat but with non-processed meat instead of white meat  As for the analysis on red meat | Selected Cross et al., 2007 with more colorectal cancer cases  (results on colon and rectal cancer from article were included in the meta-analyses)  Results on PC and DC, and on unprocessed red meat were for the highest versus lowest comparison only; included in the higher versus lower meta-analyses |
| --- | --- | --- | --- | --- | --- | --- | --- | --- |
| Spencer et al., 2010  UK Dietary Cohort Consortium [42]  (nested case-control study; pooled data from EPIC-Norfolk, EPIC-Oxford, Guernsey Study, MRC National Survey of Health and Development, Oxford Vegetarian Study, UK Women’s Cohort Study, Whitehall II) | 2575 men and women  579 cases  1996 controls  Four – seven-day food diaries | Red meat (beef, pork, lamb and meat from burgers and other non-processed meat items made with these meats)  Processed meat (ham, bacon, the meat component of sausages and other items made with processed meat)  Red and processed meat, combined |  | 579 CRC  380 CC  199 RC | 50g/day | - 1. (0.84-1.22)   2. (0.83-1.31)   0.96 (0.70-1.31)  0.88 (0.68-1.15)  1.01 (0.73-1.40)  0.65 (0.40-1.04)  0.97 (0.84-1.12)  1.03 (0.86-1.24)  0.85 (0.66-1.10) | Age, height, weight, smoking, intakes of energy, alcohol and dietary fibre; age, sex and recruitment date matched | Data from the EPIC-Norfolk and the EPIC-Oxford in this pooled analysis overlapped with the EPIC data that were already included in the meta-analysis |

* CRC – colorectal cancer; CC – colon cancer; RC – rectal cancer; PC – proximal colon cancer; DC – distal colon cancer; BMI – body mass index; FFQ – food frequency questionnaire
