# Supplementary material for: Red and Processed Meat and Colorectal Cancer Incidence: Meta-Analysis of Prospective Studies
Source: PLoS One. 2011 Jun 6;6(6):e20456. doi: 10.1371/journal.pone.0020456 (PMC3108955; doi:10.1371/journal.pone.0020456)
Supplement: Text S1 — Search strategy for the WCRF/AICR Continuous Update Project. (DOC) [file pone.0020456.s003.doc]

Text S1. Search strategy for the WCRF/AICR Continuous Update Project.

We used the standard search strategy for systematic literature review in PubMed

developed by the World Cancer Research Fund. This standard search uses a combination of subject heading terms e.g. MeSH in PubMed and has been structured to include all the exposures cited in the SLR Specification Manual (see below). This search strategy will be combined with the following search questions:

**#1** Colorectal neoplasms [MeSH] OR intestinal polyps [MeSH]

**#2** malign* [tiab] OR neoplasm* [tiab] OR carcinoma* [tiab] OR cancer* [tiab] OR tumor* [tiab] OR tumour* [tiab] OR polyp* [tiab]

**#3** colon [tiab] OR rectum [tiab] OR rectal [tiab] OR colorectum [tiab] OR colorectal [tiab] OR large bowel [tiab] OR large intestine [tiab] OR gut [tiab]

**#4** #1 OR (#2 AND #3)

Search strategy from the WCRF SLR Specification Manual (version 15) (<http://www.dietandcancerreport.org/?p=slr_specification_manual>)

**#1** diet therapy[MeSH Terms] OR nutrition[MeSH Terms]

**#2** diet[tiab] OR diets[tiab] OR dietetic[tiab] OR dietary[tiab] OR eating[tiab] OR

intake[tiab] OR nutrient*[tiab] OR nutrition[tiab] OR vegetarian*[tiab] OR vegan*[tiab]

OR "seventh day adventist"[tiab] OR macrobiotic[tiab] OR breastfeed*[tiab] OR breast

feed*[tiab] OR breastfed[tiab] OR breast fed[tiab] OR breastmilk[tiab] OR breast

milk[tiab]

**#3** food and beverages[MeSH Terms]

**#4** food*[tiab] OR cereal*[tiab] OR grain*[tiab] OR granary[tiab] OR wholegrain[tiab] OR wholewheat[tiab] OR roots[tiab] OR plantain*[tiab] OR tuber[tiab] OR tubers[tiab] OR vegetable*[tiab] OR fruit*[tiab] OR pulses[tiab] OR beans[tiab] OR lentils[tiab] OR chickpeas[tiab] OR legume*[tiab] OR soy[tiab] OR soya[tiab] OR nut[tiab] OR nuts[tiab] OR peanut*[tiab] OR groundnut*[tiab] OR seeds[tiab] OR meat[tiab] OR beef[tiab] OR pork[tiab] OR lamb[tiab] OR poultry[tiab] OR chicken[tiab] OR turkey[tiab] OR duck[tiab] OR fish[tiab] OR fat[tiab] OR fats[tiab] OR fatty[tiab] OR egg[tiab] OR eggs[tiab] OR bread[tiab] OR oils[tiab] OR shellfish[tiab] OR seafood[tiab] OR sugar[tiab] OR syrup[tiab] OR dairy[tiab] OR milk[tiab] OR herbs[tiab] OR spices[tiab] OR chilli[tiab] OR chillis[tiab] OR pepper*[tiab] OR condiments[tiab]

**#5** fluid intake[tiab] OR water[tiab] OR drinks[tiab] OR drinking[tiab] OR tea[tiab]

OR coffee[tiab] OR caffeine[tiab] OR juice[tiab] OR beer[tiab] OR spirits[tiab] OR

liquor[tiab] OR wine[tiab] OR alcohol[tiab] OR alcoholic[tiab] OR beverage*[tiab] OR

ethanol[tiab] OR yerba mate[tiab] OR ilex paraguariensis[tiab]

**#6** pesticides[MeSH Terms] OR fertilizers[MeSH Terms] OR "veterinary

drugs"[MeSH Terms]

**#7** pesticide*[tiab] OR herbicide*[tiab] OR DDT[tiab] OR fertiliser*[tiab] OR

fertilizer*[tiab] OR organic[tiab] OR contaminants[tiab] OR contaminate*[tiab] OR

veterinary drug*[tiab] OR polychlorinated dibenzofuran*[tiab] OR PCDF*[tiab] OR

polychlorinated dibenzodioxin*[tiab] OR PCDD*[tiab] OR polychlorinated biphenyl*[tiab] OR PCB*[tiab] OR cadmium[tiab] OR arsenic[tiab] OR chlorinated hydrocarbon*[tiab] OR microbial contamination*[tiab]

**#8** food preservation[MeSH Terms]

**#9** mycotoxin*[tiab] OR aflatoxin*[tiab] OR pickled[tiab] OR bottled[tiab] OR bottling[tiab] OR canned[tiab] OR canning[tiab] OR vacuum pack*[tiab] OR refrigerate*[tiab] OR refrigeration[tiab] OR cured[tiab] OR smoked[tiab] OR preserved[tiab] OR preservatives[tiab] OR nitrosamine[tiab] OR hydrogenation[tiab] OR fortified[tiab] OR additive*[tiab] OR colouring*[tiab] OR coloring*[tiab] OR flavouring*[tiab] OR flavoring*[tiab] OR nitrates[tiab] OR nitrites[tiab] OR solvent[tiab] OR solvents[tiab] OR ferment*[tiab] OR processed[tiab] OR antioxidant*[tiab] OR genetic modif*[tiab] OR genetically modif*[tiab] OR vinyl chloride[tiab] OR packaging[tiab] OR labelling[tiab] OR phthalates[tiab]

**#10** cookery[MeSH Terms]

**#11** cooking[tiab] OR cooked[tiab] OR grill[tiab] OR grilled[tiab] OR fried[tiab] OR

fry[tiab] OR roast[tiab] OR bake[tiab] OR baked[tiab] OR stewing[tiab] OR stewed[tiab] OR casserol*[tiab] OR broil[tiab] OR broiled[tiab] OR boiled[tiab] OR microwave[tiab] OR

microwaved[tiab] OR re-heating[tiab] OR reheating[tiab] OR heating[tiab] OR re heated[tiab] OR heated[tiab] OR poach[tiab] OR poached[tiab] OR steamed[tiab] OR barbecue*[tiab] OR chargrill*[tiab] OR heterocyclic amines[tiab] OR polycyclic aromatic hydrocarbons[tiab]

**#12** dietary carbohydrates[MeSH Terms] OR dietary proteins[MeSH Terms] OR sweetening agents[MeSH Terms]

**#13** salt[tiab] OR salting[tiab] OR salted[tiab] OR fiber[tiab] OR fibre[tiab] OR

polysaccharide*[tiab] OR starch[tiab] OR starchy[tiab] OR carbohydrate*[tiab] OR

lipid*[tiab] OR linoleic acid*[tiab] OR sterols[tiab] OR stanols[tiab] OR sugar*[tiab] OR

sweetener*[tiab] OR saccharin*[tiab] OR aspartame[tiab] OR acesulfame[tiab] OR

cyclamates[tiab] OR maltose[tiab] OR mannitol[tiab] OR sorbitol[tiab] OR sucrose[tiab]

OR xylitol[tiab] OR cholesterol[tiab] OR protein[tiab] OR proteins[tiab] OR hydrogenated dietary oils[tiab] OR hydrogenated lard[tiab] OR hydrogenated oils[tiab]

**#14** vitamins[MeSH Terms]

**#15** supplements[tiab] OR supplement[tiab] OR vitamin*[tiab] OR retinol[tiab] OR

carotenoid*[tiab] OR tocopherol[tiab] OR folate*[tiab] OR folic acid[tiab] OR methionine[tiab] OR riboflavin[tiab] OR thiamine[tiab] OR niacin[tiab] OR pyridoxine[tiab] OR cobalamin[tiab] OR mineral*[tiab] OR sodium[tiab] OR iron[tiab] OR calcium[tiab] OR selenium[tiab] OR iodine[tiab] OR magnesium[tiab] OR potassium[tiab] OR zinc[tiab] OR copper[tiab] OR phosphorus[tiab] OR manganese[tiab] OR chromium[tiab] OR phytochemical[tiab] OR allium[tiab] OR isothiocyanate*[tiab] OR glucosinolate*[tiab] OR indoles[tiab] OR polyphenol*[tiab] OR phytoestrogen*[tiab] OR genistein[tiab] OR saponin*[tiab] OR coumarin*[tiab]

**#16** physical fitness[MeSH Terms] OR exertion[MeSH Terms] OR physical endurance[MeSH Terms] or walking[MeSH Terms]

**#17** recreational activit*[tiab] OR household activit*[tiab] OR occupational activit*[tiab] OR physical activit*[tiab] OR physical inactivit*[tiab] OR exercise[tiab]

OR exercising[tiab] OR energy intake[tiab] OR energy expenditure[tiab] OR energy

balance[tiab] OR energy density[tiab]

**#18** growth[MeSH Terms] OR anthropometry[MeSH Terms] OR body composition[MeSH Terms] OR body constitution[MeSH Terms]

**#19** weight loss[tiab] or weight gain[tiab] OR anthropometry[tiab] OR birth

weight[tiab] OR birthweight[tiab] OR birth-weight[tiab] OR child development[tiab] OR

height[tiab] OR body composition[tiab] OR body mass[tiab] OR BMI[tiab] OR obesity[tiab] OR obese[tiab] OR overweight[tiab] OR over-weight[tiab] OR over weight[tiab] OR skinfold measurement*[tiab] OR skinfold thickness[tiab] OR DEXA[tiab] OR bio-impedence[tiab] OR waist circumference[tiab] OR hip circumference[tiab] OR waist hip ratio*[tiab]

**#20** #1 OR #2 OR #3 OR #4 OR #5 OR #6 OR #7 OR #8 OR #9 OR #10 OR #11 OR

#12 OR #13 OR #14 OR #15 OR #16 OR #17 OR #18 OR #19

**Optional:**

**#21** animal[MeSH Terms] NOT human[MeSH Terms]

**#22** #20 NOT #21
